# Endothelial tyrosine kinase receptor B prevents VE-cadherin cleavage and protects against atherosclerotic lesion development in ApoE−/− mice

**DOI:** 10.18632/oncotarget.5855

**Published:** 2015-09-28

**Authors:** Hong Jiang, Shuhong Huang, Xinyun Li, Xian Li, Shanying Huang, Yun Zhang, Zhe-Yu Chen

**Affiliations:** ^1^ Key Laboratory of Cardiovascular Remodeling and Function Research, Chinese Ministry of Education and Chinese Ministry of Health, Qilu Hospital, Shandong University, Jinan, Shandong, China; ^2^ Department of Neurobiology, Shandong Provincial Key Laboratory of Mental Disorders, School of Medicine, Shandong University, Jinan, Shandong, China

**Keywords:** atherosclerosis, endothelial barrier dysfunction, brain-derived neurotrophic factor, tyrosine kinase receptor B, vascular endothelial cadherin, Pathology Section

## Abstract

Tyrosine kinase receptor B (TrkB) is a high-affinity receptor for brain-derived neurotrophic factor (BDNF). In addition to its nervous system functions, TrkB is also expressed in the aortic endothelium. However, the effects of endothelial TrkB signaling on atherosclerosis remained unknown. Immunofluorescence analysis revealed that TrkB expression is downregulated in the endothelium of atherosclerotic lesions from ApoE−/− mice compared with the atheroma-free aorta of WT mice. Endothelial TrkB knockdown led to increased lesion size, lipid deposition and inflammatory responses in the atherosclerotic lesions of the ApoE−/− mice compared with the control mice. Mechanistic studies showed that TrkB activation prevented VE-cadherin shedding by enhancing the interaction between vascular endothelial protein tyrosine phosphatase and VE-cadherin, maintaining VE-cadherin in a dephosphorylated state. Our data demonstrate that TrkB is an endothelial injury-response molecule in atherogenesis. Endothelial BDNF/TrkB signaling reduces VE-cadherin shedding and protects against atherosclerotic lesion development in ApoE−/− mice.

## INTRODUCTION

Tyrosine kinase receptor B (TrkB) is a high-affinity receptor for brain-derived neurotrophic factor (BDNF). The BDNF/TrkB pathway plays critical roles in the survival, growth, and maintenance of central and peripheral neurons [1]. In addition to its nervous system functions, BDNF/TrkB pathway also functions in the cardiovascular system. The BDNF/TrkB pathway has been reported to protect the myocardium against ischemic injury [2]. Constitutive BDNF/TrkB signaling is required for normal cardiac contraction and relaxation [3]. We and other researchers have reported that low plasma levels of BDNF associated with increased risk of cardiovascular disease [4–7]. BDNF deficiency results in endothelial cell apoptosis, intraventricular wall hemorrhage, depressed cardiac contractility and early postnatal death in mice [8]. Mice with a disrupted TrkB gene lack a significant proportion of intramyocardial blood vessels and showed early postnatal death [9]. We recently reported that TrkB was prominently expressed in the endothelium of atherosclerotic lesions and maintained endothelial barrier integrity by regulating VE-cadherin expression [10]. However, the association between endothelial TrkB signaling and atherosclerosis has not been determined.

In this study, we compared TrkB expression in aortas with and without atherosclerotic lesions in mice and investigated atherosclerotic lesion size, lipid and macrophage components, proinflammatory markers expression in ApoE−/− mice after knocking down endothelial TrkB, seeking to reveal the role and mechanism of endothelial TrkB signaling in the development of atherosclerotic lesions.

## RESULTS

### TrkB expression in the endothelium is downregulated in atherosclerotic lesions of ApoE−/− mice compared with the atheroma-free aorta of WT mice

**Figure 1:**
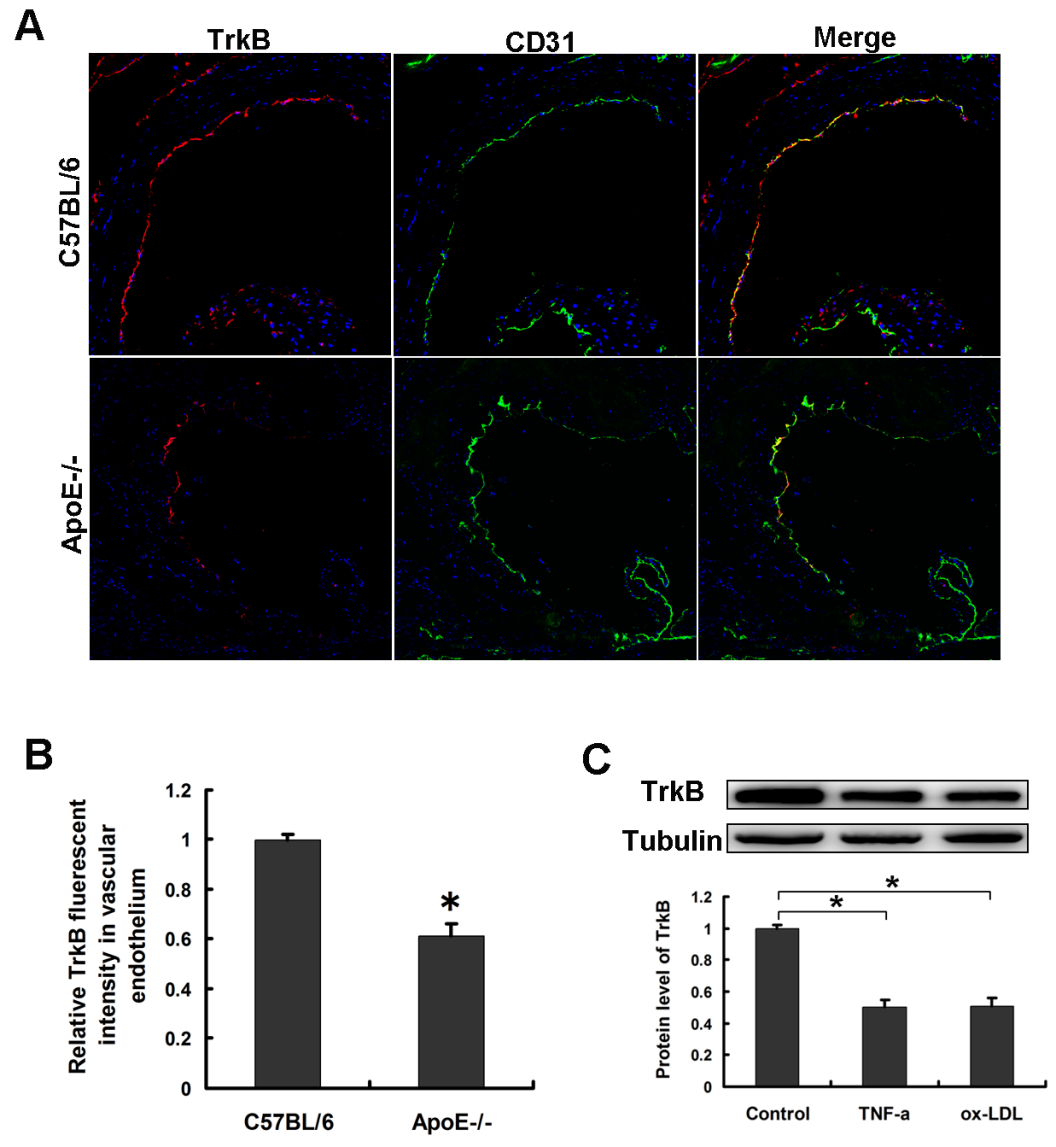
The expression of TrkB in endothelial cells was downregulated under atherosclerotic states **A.**, **B.** The expression of TrkB in atherosclerotic lesions of ApoE−/− mice was downregulated. **A.** Aortic root sections of WT (*n* = 5) or ApoE−/− (*n* = 5) mice fed a high-cholesterol diet for 8 weeks were subjected to immunofluorescence staining for TrkB and CD31. **B.** The relative fluorescent intensity of TrkB in the endothelium of aortic roots from WT and ApoE−/− mice (*n* = 5 sections per tissue, at least 3 analysis sites per slide). *P < 0.05 vs. WT mice. **C.** TrkB expression in HAECs was decreased after stimulation with 50ng/mL TNF-α or 40μg/ml ox-LDL for 12 hours.

We performed immunofluorescence staining to compare the expression of TrkB in C57BL/6 mice (controls) and ApoE−/− mice fed with an atherogenic diet for 8 weeks. In agreement with previous results [10], TrkB expression was prominent in the aortic ECs of atherosclerotic lesions. Importantly, the relative fluorescent intensity analysis demonstrated that the TrkB expression observed in the endothelium was decreased by 35% in the atheroma of ApoE−/− mice compared with the atheroma-free aorta of WT mice (Figure 1A, 1B). Considering that atherosclerosis is an inflammatory disease, then we investigated the effects of proinflammatory factors on TrkB expression in HAECs by administration with or without TNF-α, ox-LDL. Western blot analysis demonstrated that TrkB expressions in HAECs were significantly inhibited after TNF-α or ox-LDL administration (Figure 1C), which suggested that inflammatory factors might downregulate endothelial TrkB levels in atherosclerotic lesions.

### Endothelial TrkB confers atheroprotection in ApoE−/− mice

**Figure 2:**
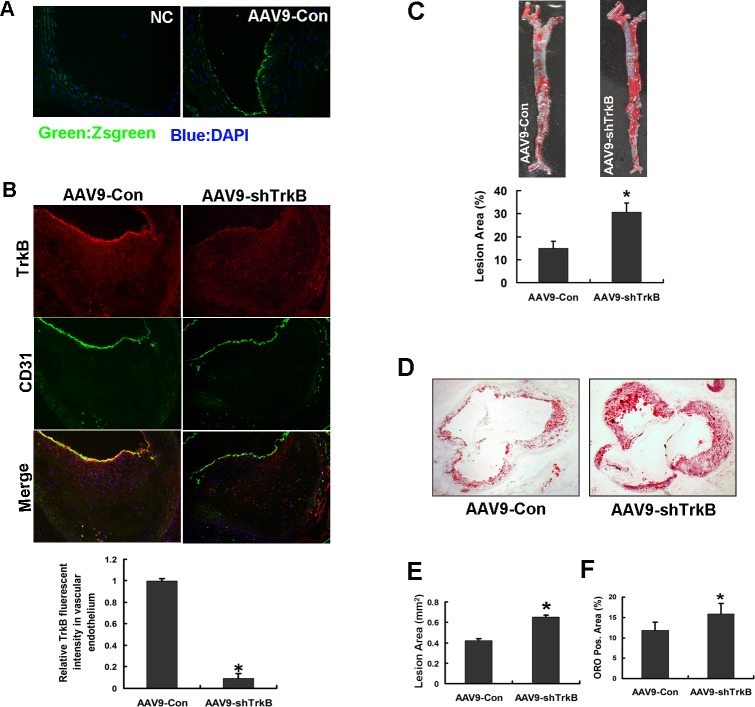
Endothelial TrkB confers atheroprotection in apoE−/− mice Eight-week-old male ApoE−/− mice (*n* = 10/group) were administered with AAV9-Con or AAV9-shTrkB via the tail vein, followed by feeding of a high-cholesterol diet for 12 weeks. **A.** The highly efficient expression of the reporter gene Zsgreen, carried by AAV9, in the aortic endothelial cells of the lesion. **B.** Double immunfluorescence staining for TrkB and CD31 and relative fluorescent intensity of TrkB in the endothelium of aortic roots from ApoE−/− mice infected with AAV9-control or AAV9-shTrkB (*n* = 5 sections per tissue, at least 3 analysis sites per slide). **C.** En face staining of lesion areas with Oil Red O in the aorta. The data represent the percentage surface area of the aorta occupied by lesions in the ApoE−/− mice with or without TrkB knockdown. *P < 0.05. **D.** Representative photographs of a cross section of the aortic sinus stained with Oil Red O in the mice. **E.** The lesion sizes of the intimal area were measured using ImagePro-Plus. **F.** Quantitative computer-assisted image analysis of the lesions for lipid deposition. *P < 0.05.

Our previous study revealed that TrkB maintained endothelial barrier integrity [10]. Then the effects of endothelial TrkB knockdown on atherosclerosis were evaluated. ApoE−/− mice were systemically infected with Adeno-associated virus serotype-9 (AAV9) carrying a Zsgreen reporter gene (AAV9-control) or AAV9 carrying small hairpin RNA-TrkB (AAV9-shTrkB) via the tail vein and fed with an atherogenic diet for 12 weeks. After systemic infection, highly efficient expression of the reporter gene Zsgreen was observed in the endothelial layer of atherosclerotic lesions in ApoE−/− mice (Figure 2A). Immunofluorescence staining revealed a significant 91% reduction of TrkB expression in the aorta of the ApoE−/− mice infected with AAV9-shTrkB compared with the control mice with AAV9-Control infection (Figure 2B), suggesting that the levels of TrkB in the aortic endothelial layer was efficiently knocked down. The introduction of AAV9-shTrkB into apoE−/− mice significantly increased the lesion area in aortic trees compared with the introduction of AAV9-control (Figure 2C). Similar results were also found in the intimal area of aortic sinus cross-sections from the mice (Figure 2D, 2E). These mice displayed no change in food consumption and weight. The introduction of the two types of AAV9 did not significantly change the plasma concentration of lipids, including triglycerides and total, LDL, and high-density lipoprotein (Table 1). Our data revealed that endothelial TrkB confers atheroprotection in apoE−/− mice.

**Table 1:** Plasmid lipid contents of ApoE−/− mice transfected with AAV9-control, AAV9-shTrkB fed HCD for 12 weeks

Lipid (mol/L)	AAV9-control (n=10)	AAV9-shTrkB (n=10)	*P*
Total cholesterol Triglyceride LDL HDL	23.9±6.6 1.14±0.58 2.43±0.42 6.33±0.71	23.6±5.7 1.23±0.67 2.47±0.81 6.26±0.84	NS NS NS NS

### Endothelial TrkB knockdown leads to increased lipid deposition, macrophage infiltration and inflammatory responses in the atherosclerotic lesions of ApoE−/− mice

**Figure 3:**
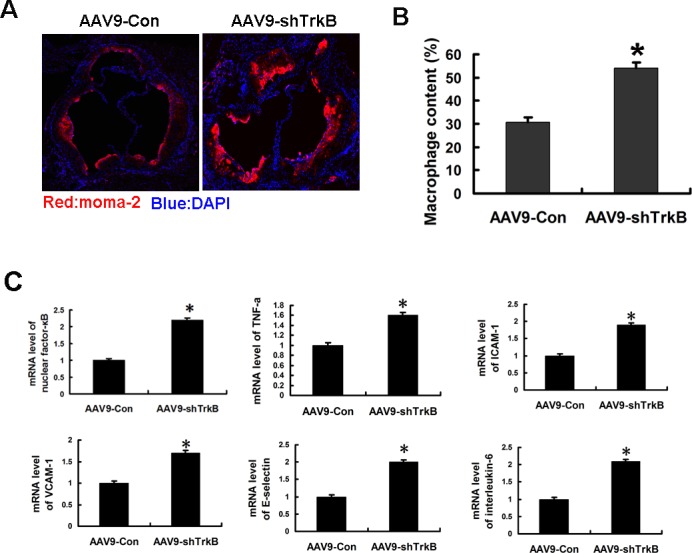
Increased macrophage infiltration and mRNA induction of proinflammatory markers in the atherosclerotic lesions of ApoE−/− mice by TrkB knockdown **A.**, **B.** Representative photographs of a cross section of the aortic sinus stained with moma-2. The macrophage contents in the lesions of the aortic sinus were measured. *P < 0.05. **C.** Real-time PCR showed the mRNA levels of nuclear factor-κB, intercellular adhesion molecule-1, vascular cell adhesion molecule-1, E-selectin, tumor necrosis factor-α, and interleukin-6 in the aortas of the mice. *P < 0.05.

Then the lipid and macrophage deposition in the atherosclerotic lesions of ApoE−/− mice was evaluated. Lipid deposition in the lesion, as demonstrated by the Oil Red O-positive region, was significantly increased in ApoE−/− mice infected with AAV9-shTrkB (Figure 2F). The infiltration of macrophages into the vascular wall, as assessed by immunofluorescence with MOMA-2 antibody, was significantly increased with the AAV9-shTrkB (Figure 3A, 3B). Next, we investigated the inflammatory responses in the lesions. Real-time PCR showed that the mRNA levels of the proinflammatory markers, including nuclear factor-κB, intercellular adhesion molecule-1, vascular cell adhesion molecule-1, E-selectin, tumor necrosis factor-α, and interleukin-6, in the aortas of ApoE−/− mice were significantly increased in the TrkB knockdown group compared with those in control group (Figure 3C). Our data revealed that TrkB protects aortas against lipoprotein leakage and monocyte extravasation and inflammatory responses during atherogenesis in ApoE−/− mice.

### BDNF prevented TNF-α-induced-shedding of VE-cadherin in HAECs

**Figure 4:**
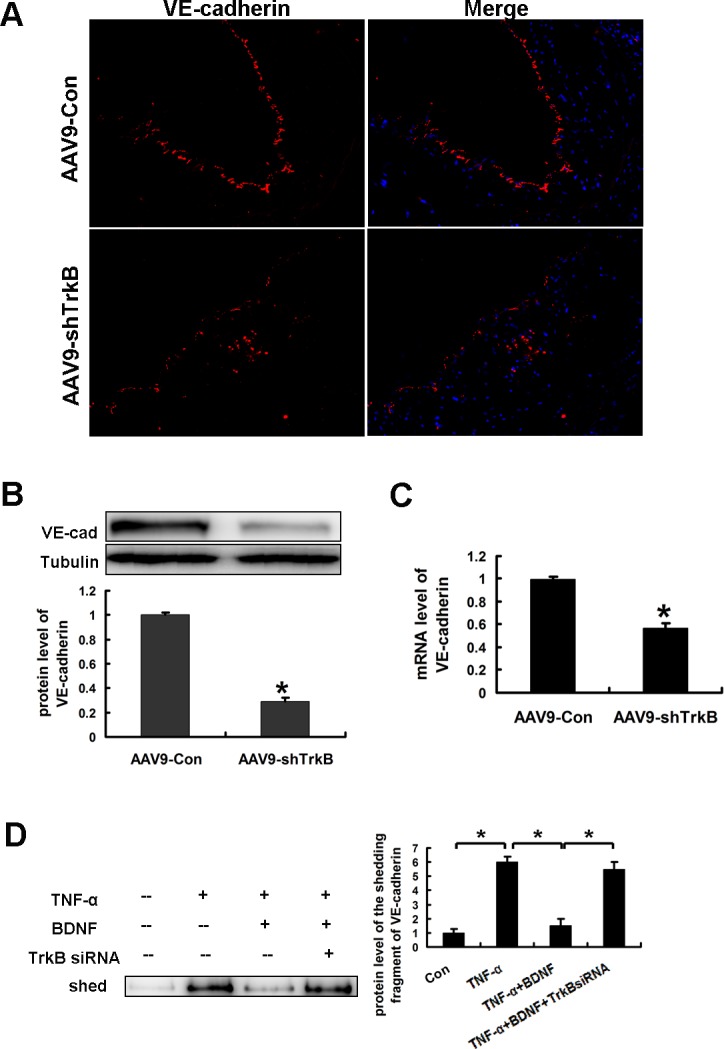
BDNF prevented TNF-α induced-shedding of VE-cadherin in HAECs **A.** The expression of VE-cadherin in the endothelium in aortic lesions from the ApoE−/− mice with or without TrkB knockdown is shown by immunofluorescence staining. **B.** Real-time PCR and **C.** western blot analysis showed the mRNA and protein levels, respectively, of VE-cadherin in the aortas of the mice. **D.** The medium of HAECs treated as indicated was immunoprecipitated with anti-VE-cadherin antibody targeting its extracellular fragment and the immunoprecipitates were then examined for the shedding fragment using western blot analysis.

Our previous study revealed that TrkB promoted VE-cadherin synthesis in HAECs [10]. As expected, immunofluorescence staining showed that the VE-cadherin expression in atherosclerotic lesions was reduced by TrkB knockdown (Figure 4A). Importantly, by comparing the mRNA and protein levels of VE-cadherin after TrkB knockdown, we found that VE-cadherin protein was decreased by 79%, whereas the VE-cadherin mRNA was only decreased by 43% (Figure 4B, 4C). These data suggested that TrkB might be involved in both the VE-cadherin synthesis and cleavage.

It has been reported that an extracellular fragment of VE-cadherin can be cleaved and detected in medium [14]. Then we investigated whether BDNF-TrkB inhibited the shedding of VE-cadherin. As shown in Figure 4D, we found a 90KD shedding fragment of VE-cadherin in the medium consistent with previous study [15]. Importantly, the shedding fragment of VE-cadherin were significantly reduced in the medium of TNF-α-stimulated HAECs by preincubation with BDNF, and the effects were blocked by TrkB siRNA, suggesting that BDNF prevented TNF-α- induced shedding of VE-cadherin by TrkB activation.

### BDNF regulated tyrosine phosphorylation of VE-cadherin and binding of VE-cadherin and VE-PTP in HAECs

**Figure 5:**
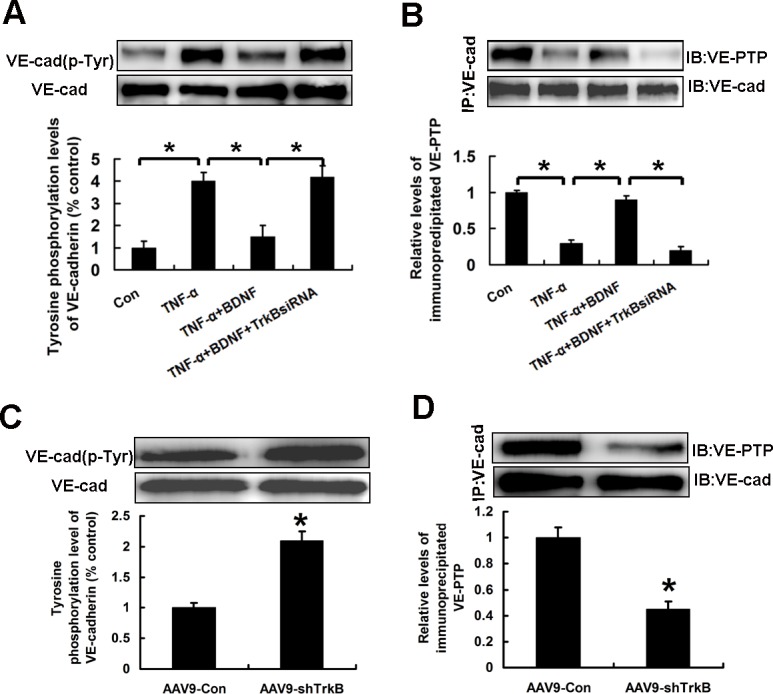
BDNF regulated tyrosine phosphorylation of VE-cadherin and binding of VE-cadherin and VE-PTP in HAECs **A.**, **B.** The cells extracts of HAECs treated as indicated were immunoprecipitated with VE-cadherin antibody and the immunoprecipitates were then examined using the **A.** anti-Phospho-Tyr antibody or **B.** anti-VE-PTP antibody. **C.**, **D.** The extracts of aortas in ApoE−/− mice were immunoprecipitated with VE-cadherin antibody and the immunoprecipitates were then examined using the **C.** anti-Phospho-Tyr antibody or **D.** anti-VE-PTP antibody. *P < 0.05.

Tyrosine phosphorylation processes of VE-cadherin are required for the VE-cadherin cleavage [16]. Then, we investigated the effects of BDNF on the tyrosine phosphorylation levels of VE-cadherin. As reported by Sidibé et al [16], we also found that TNF-α significantly induced the tyrosine phosphorylation of VE-cadherin. Importantly, BDNF reduced the tyrosine phosphorylation levels of VE-cadherin induced by TNF-α, and the effects were blocked by TrkB siRNA (Figure 5A).

As a transmembrane binding partner of VE-cadherin, vascular endothelial protein tyrosine phosphatase (VE-PTP) binds directly to VE-cadherin. The binding maintains VE-cadherin in a dephosphorylated state [17]. Then, we evaluated whether BDNF promoted the binding of VE-PTP and VE-cadherin. The cells extracts of HAECs were immunoprecipitated with VE-cadherin antibody. As shown in Figure 5B, the levels of the immunoprecipitated VE-PTP were significantly higher in HAECs treated with BDNF, and the effects were blocked by TrkB siRNA. Similarly, the aortas of ApoE−/− mice with TrkB knockdown also displayed increased tyrosine phosphorylation of VE-cadherin and reduced binding of VE-PTP and VE-cadherin compared with control mice (Figure 5C, 5D).

## DISCUSSION

In this study, we investigated the role of endothelial TrkB signals in the development of atherosclerotic lesion in ApoE−/− mice. Our data showed that TrkB expression in the endothelium was downregulated in atherosclerotic lesions. Endothelial TrkB knockdown led to increased lesion size, lipid deposition and inflammatory responses in the atherosclerotic lesions of the ApoE−/− mice compared with the control mice. Finally, TrkB activation prevented the VE-cadherin shedding by maintaining the interaction between VE-PTP and VE-cadherin and reducing tyrosine phosphorylation of VE-cadherin. Our data supported that endothelial TrkB signaling may be a novel therapeutic target for atherosclerosis.

TrkB is a high-affinity receptor for BDNF with well-established functions in the nervous system. However, TrkB is also expressed in the aortic endothelium. In this study, we found that TrkB expression in the endothelium to be downregulated in atherosclerotic lesions of ApoE−/− mice compared with the atheroma-free aorta of WT mice. *In vitro* experiments we further demonstrated that the TrkB levels in HAECs were significantly decreased by proinflammatory factors. Our data suggest that TrkB is an endothelial injury-response molecule in atherogenesis and TrkB may participate in the pathologic process of atherosclerosis. Consistently, the decreased BDNF/TrkB levels were also found in the tissues of aged or patients with CAD-related complications. Decreased expression of BDNF in the endothelium was associated with hypertension [18]. In human umbilical vein endothelial cells the expression of BDNF was downregulated by laminar fluid shear stress [19]. In the hippocampus and temporal cortex, TrkB mRNA levels decreased with age [20]. The reason of reduced TrkB expression in injury-tissues requires further investigations.

In this study, we revealed a novel role of endothelial TrkB in cardiovascular system as an atheroprotector. Our data demonstrated that endothelial BDNF-TrkB pathway deficiency led to accelerated development of atherosclerotic lesions in the aortic trees and aortic root of ApoE−/− mice. Similarly, as a growth factor signal, angiopoietin-1 has been reported to protect adult vasculature from leakage [21]. The administration of adenoviral Ang-2 to apoE−/− mice fed a Western diet significantly reduced the oxidized LDL and macrophage content of the plaques [22]. Therefore, our data revealed that the endothelial BDNF-TrkB pathway is a novel growth factor signal for protecting against atherosclerosis. Our previous study revealed that TrkB maintained endothelial barrier integrity by promoting VE-cadherin expression [10]. In this study, our data further confirmed that TrkB protects aortas against lipoprotein leakage and monocyte extravasation and inflammatory responses during atherogenesis in ApoE−/− mice. The VE-cadherin levels in the lesions of ApoE−/− mice with TrkB knockdown were also reduced. Therefore, the protective role of endothelia TrkB against atherosclerosis may be mainly mediated by maintaining VE-cadherin-dependent endothelial barrier function. However, our data appear to be in conflict with the results of Kraemer et al [23]. In Kraemer's paper, there was a 30% to 40% reduction in lesion size, witch caused by decreased smooth muscle cell accumulation, in TrkB+/− mice compared with TrkB+/+ mice. The reason may be that the expression of TrkB in the smooth muscle cells was not knockdowned in our study, because we don't observe the reporter gene expression of AAV9 vectors in smooth muscle cells of the aorta. Then the increased lesion size, lipid deposition and inflammatory responses are just from the endothelial TrkB knockdown in our study. Consistently, Kraemer also found macrophage immunoreactivity to be increased from 30% to 49% of the total lesion area in TrkB+/− mice. Therefore, TrkB may stabilize atherosclerotic plaque via simultaneously maintaining endothelial barrier integrity and promoting smooth muscle cell activity.

In this study we found that BDNF/TrkB signaling prevented the TNF-α induced-VE-cadherin shedding. The elevated shedding fragment of VE-cadherin has been found to be associated with diabetic retinopathy and coronary atherosclerosis [15, 24]. Thus the downregulation of TrkB in the endothelium in atherosclerotic lesions may be an underlying reason for elevated shedding fragments of VE-cadherin in patients with diabetic retinopathy or coronary atherosclerosis. We further elucidate the mechanism by which TrkB preventing the TNF-α induced-VE-cadherin shedding. Our data demonstrated that BDNF/TrkB signaling involved in the tyrosine phosphorylation of VE-cadherin and the interaction between VE-PTP and VE-cadherin, leading to reduced endothelial cell-cell dissociation and hyperpermeability [25]. Therefore, our results provided a novel downstream molecular signaling of TrkB in protecting endothelial barrier integrity. How BDNF/TrkB regulates the interaction between VE-PTP and VE-cadherin is not clear and further research should be performed to elucidate these mechanisms.

Our data demonstrate that TrkB expression in the endothelium is downregulated in the atherosclerotic lesions of ApoE−/− mice. In addition to promoting the synthesis of VE-cadherin, endothelial BDNF/TrkB signaling also regulates the shedding of VE-cadherin and protects against atherosclerotic lesion development in ApoE−/− mice. Endothelial TrkB may be used as a novel therapeutic target in atherosclerosis.

## MATERIALS AND METHODS

### Animal study protocol

All procedures were performed in accordance with the Guide for the Care and Use of Laboratory Animals published by the U.S. National Institutes of Health (NIH Publication No.85-23, revised 1996) and were approved by the Ethics Committee of Shandong University. Eight-week-old male ApoE−/− mice received a single systemic administration of 2.1×10^11^ vg/g body weight particles of adeno-associated virus serotype-9 (AAV9) with or without shRNA specifically targeting TrkB via the tail vein [11]. For TrkB knockdown, the shRNA sequences were 5′-gaa cat caa gag cat cca ctt caa gag agt gga tgc tct tga tgt tct ttt tt-3′ and 5′-aaa aaa gaa cat caa gag cat cca ctc tct tga agt gga tgc tct tga tgt tc-3′. To induce atherosclerotic lesion development, the ApoE−/− mice were fed a high-cholesterol diet containing 42% fat and 0.2% cholesterol for 12 weeks. After overnight fasting, blood was collected via heart puncture for the measurement of plasma lipids. Then, the aortas were harvested for Oil Red O staining, immunofluorescence staining, and molecular biology analyses.

### Oil red O staining

Oil Red O staining was used to assess the size of the atherosclerotic lesion as previously described [12]. Briefly, the aortic trees and cross-sections of the aortic sinuses were fixed with 4% paraformaldehyde. After washing with PBS, the aorta was opened longitudinally and pinned flat on a black surface. Then, the aortic trees and cross-sections of the aortic sinuses were stained with Oil Red O (Sigma, USA). For quantification, the lesion size of the aorta and the intimal areas of the aortic sinuses were measured using the ImagePro-Plus software.

### Immunofluorescence analysis

Immunofluorescence staining was used to show the expression of TrkB and VE-cadherin, and macrophage content of the lesions. Briefly, the cryosections of the aortic sinus were fixed with 4% paraformaldehyde. After washing with PBS and blocking with serum, the sections were incubated with anti-TrkB (sc-12), anti-CD31 (Abcam), anti-VE-cadherin (C-19; Santa Cruz), or anti-mouse macrophage (moma-2, AbD) antibodies. Then, the FITC or TRITC-conjugated secondary antibodies were used. Finally, the sections were observed using fluorescence microscopy.

### Western blot analysis

Antibodies for anti-TrkB (sc-12), anti-VE-cadherin (C-19), and anti-vascular endothelial protein tyrosine phosphatase (sc-65228) were purchased from Santa Cruz. The preparation of the cell lysates, measurement of the protein concentrations, sodium dodecyl sulfate-polyacrylamide gel electrophoresis (SDS-PAGE), electrophoretic transfer, sequential incubation with primary and second antibodies and film development were performed as previously described [13].

### Real-time PCR

Total RNA was isolated from the aortas of the mice. First cDNA was synthesized using AMV reverse transcriptase. PCR was performed using the following primers: forward, 5′-AGC AGG GAA ACA TCT ATA ACG-3′ and reverse, 5′-CTT GAA CTT TGG GTT TAC TGG-3′ for mouse VE-cadherin; 5′-AGG CTT CTG GGC CTT ATG TG-3′ and 5′-GTA TTC CTG GCG AGA GAA GCA-3′ for NF-κB; 5′-GTG AAG GGA ATG GGT GTT-3′ and 5′-GGT CAC TGT CCC AGC ATC-3′ for TNF-α; 5′-TTC CAG AAA CCG CTA TGA-3′ and 5′-GGT TGT CAC CAG CAT CAG-3′ for IL-6; 5′-GGC ACC CAG CAG AAG TTG TT-3′ and 5′-CTT GGT AGA GGT GAC TGA GG-3′ for ICAM-1; 5′-TCT CTC AGG AAA TGC CAC CC-3′ and 5′-GTG TGC TGC TAT TGG CTG TG-3′ for VCAM-1; 5′-TCC TCT GGA GAG TGG AGT GC-3′ and 5′-ATG TGA AGC TTT GAC CCA CC-3′ for E-selectin as well as 5′ -TGT CTC CTG CGA CTT CAA CA-3′ and 5′-GGT GGT CCA GGG TTT CTT ACT-3′ for mouse GAPDH. The relative expression of the genes was obtained through the 2^ΔΔCt^ calculation.

### Cell culture

Human aortic endothelial cells (HAEC) were purchased from ATCC (Manassas, VA). The HAECs were cultured in endothelial cell medium. All cells were cultured at 37°C with 5% CO2. All assays were performed in triplicate.

### Statistical analysis

The animal study data are presented as the mean ± SEM. For comparisons between two groups, Student's t test was employed. All statistical tests were 2-tailed with *P* < 0.05 set as the significance level, and they were performed using the SPSS 15.0 software (SPSS, Chicago, IL).
